# Perceptions of Good Health and Impact of COVID-19 Among Adolescents in a Low-Income Urban Agglomerate in Delhi, India: A Qualitative Study

**DOI:** 10.7759/cureus.24425

**Published:** 2022-04-23

**Authors:** Nandini Sharma, Saurav Basu, Subhanwita Manna, Shivani Rao, Pragya Sharma, Harpreet Kaur, Kushagr Duggal, Pawan Kumar, Shikha T Malik

**Affiliations:** 1 Department of Community Medicine, Maulana Azad Medical College, New Delhi, IND; 2 Indian Institute of Public Health, Public Health Foundation of India, New Delhi, IND; 3 State Health Intelligence Bureau, Directorate General of Health Services, New Delhi, IND; 4 Department of Biotechnology, National Biopharma Mission, Biotechnology Industry Research Assistance Council, New Delhi, IND

**Keywords:** covid-19, adolescent and sexual health, health service utilisation, health information seeking behavior, adolescents' health

## Abstract

Adolescents constitute 16% of the global population and are susceptible to adverse health and illness from substance abuse, unhealthy diet, physical inactivity, and high-risk sexual behaviors. We conducted this study to assess the perceptions of good health, health-seeking behavior, and health service utilization among adolescents living in a low-income urban neighborhood after the second wave of the COVID-19 pandemic. A total of 23 adolescents, including 12 males and 11 females, were interviewed. Adolescents' perceived body image and size considerations apart from functioning at an optimum physical capacity as the principal attributes of good health, which was possible through the intake of a healthy diet and exercise. Adolescents were likely to be aware of the addiction potential and risk of cancer from using tobacco and alcohol, but attitudes towards eschewing their use were ambivalent. Adolescents perceived themselves as lacking access to reliable, adequate, and validated sources of sexual and reproductive health information. Knowledge and utilization of adolescent health services in this area were negligible, suggestive of the need to strengthen these services and improve the program outreach.

## Introduction

Globally, India has the largest adolescent population, with an estimated 235 million that is expected to grow until 2050 [1]. Adolescence represents the period of cognitive, physical, and emotional transition from childhood to adulthood [2]. Adolescents are vulnerable to adverse health conditions due to their tendency for risk-taking behaviors, experimentation with addictive substances, technological addictions, sedentary lifestyles, violence and injuries, and suboptimal nutrition. However, adolescents may lack understanding and awareness of the correct health and lifestyle choices to protect themselves and may restrict interpersonal communication with parents and teachers, which may accentuate this problem [1]. Furthermore, adolescents in low-income countries are likely to experience greater health disparities due to the limited availability of healthcare services, further accentuating their risk of preventable illness. It is estimated that US$ 4.6 per capita in physical, mental, and sexual health treatments from 2015 to 2030 will result in a ten-fold increase in economic gains by reducing premature adolescent mortality [3].

Adolescent Friendly Health Services (AFHS) were established across countries in response to the health needs of adolescents. In 2014, India started the Rashtriya Kishor Swasthya Karyakram (RKSK) National Adolescent Health Program to meet the health needs of the adolescent population, but service delivery and utilization remain low [4]. A study conducted in the state of Puducherry in India reported that AFHS was used by only 15-19% of adolescent girls and none of the adolescent boys [5]. This low utilization of adolescent health services was also observed in the states of Jharkhand, Rajasthan, and Maharashtra, where only 4.5% of adolescent boys and 7.8% of adolescent girls had ever heard about these services [6]. During the COVID-19 pandemic, primary health care services were detrimentally impacted, particularly in the developing world, further worsening health care access for adolescents [7]. Moreover, COVID-19 is linked to increased stress, anxiety, and depression, which are also accentuated by prolonged school closures, reduced social interaction with peers, and worsening household poverty, factors that negatively impact adolescent health, particularly in vulnerable, low-income households [8,9].

Therefore, we conducted this study to assess the perceptions of good health, health-seeking behavior, and health service utilization among adolescents living in a low-income urban neighborhood after the second wave of the COVID-19 pandemic.

## Materials and methods

Study design and setting

We conducted a qualitative research study among adolescents residing in a low-income urban agglomerate in Delhi, India, from July 2021 to September 2021. The study area is an upcoming demographic and health surveillance site located in the northeast district of Delhi, and the participants were selected from this cohort by the simple random sampling method. A total of 23 adolescent participants willing to participate and with parental approval were included in the study, and adequate saturation was considered to have been achieved for this homogenous study population. 

Procedure

Information on sociodemographic characteristics and health-seeking behavior was collected from the participants using a structured interview schedule. In-depth unstructured face-to-face interviews were conducted using a pretested topic interview guideline to assess adolescent perspectives on health and well-being, perceived barriers to good health, trustworthy sources of health information, and their preferred health-seeking behavior for personal health problems. Each interview lasted 20-25 minutes and was conducted by a trained male and female investigator amongst male and female adolescent participants, respectively.

The study tools were originally prepared in English in consultation with a group of public health experts to ensure face validity. The English tool was then linguistically validated in Hindi, the local language, conforming to the recommended forward translation process by two individuals having native fluency in both languages. 

Conceptual framework

**Figure 1:**
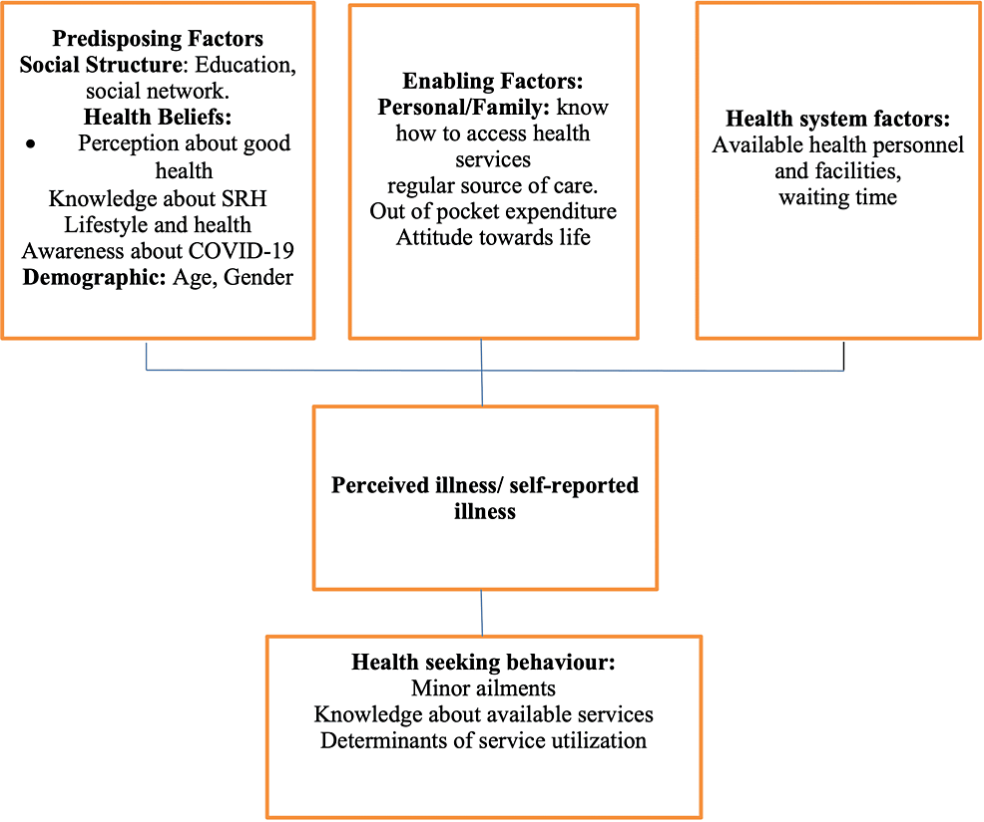
Conceptual framework for adolescent care seeking behavior Adapted from Anderson [10] SRH - sexual and reproductive health

The conceptual framework used for this study is adapted from the modified Andersen and Newman model, wherein an individual's access to and use of health care is determined by predisposing, enabling, and health-system factors [10]. Perceived severity of sickness was an additional factor incorporated from Kroeger's (1983) model [11] (Figure 1).

Data analysis

First, the verbal data from face-to-face interviews was transcribed as verbatim while referring to the data collectors' field notes. The interviews were then discussed and recorded, including the content and nonverbal expressions of the participants. Repeated readings of the transcripts and listening to the audio recordings were used to familiarize the data. The transcripts were analyzed by two investigators to systematically classify the coded data under the key conversation subjects, allowing main themes to emerge from each discussion topic. The agreement with themes was double-checked to clear differences, and the final themes were titled and defined. Acceptable transcript excerpts were selected and agreed upon to support the overall ideas.

## Results

Participant characteristics

A total of 23 participants, including 12 boys and 11 girls, were enrolled in this study. The mean age of the boys was 16.41 (SD=1.38) years, whereas the mean age of girls was 17.09 (SD=1.51) years. For treating minor ailments, boys were more likely to visit government health facilities (58.3%) compared to the girls who preferred visiting private health facilities (54.5%) (Table 1). 

**Table 1:** Sociodemographic characteristics of participants

Characteristics	Female (n=11) N, %	Male (n=12) N, %	Total (n=23) N, %
Age (years)			
10 to 15	3 (27.3%)	4 (33.33%)	7 (30.4%)
16 to 19	8 (72.7%)	8 (66.7%)	16 (69.6%)
Height (mean, SD)	146.9, 10.72	162.9, 11.5	155.3, 13.6
Weight (mean, SD)	40.7, 5.56	52.5, 13.5	46.9, 11.9
Educational status			
Up to secondary	6 (54.5%)	4 (33.3%)	10 (43.5%)
Up to higher secondary	3 (27.3%)	6 (50.0%)	9 (39.1%)
Graduation	2 (18.2%)	0	2 (8.7%)
Vocational course	0	2 (16.7%)	2 (8.7%)
Occupation			
Student	10 (90.9%)	12 (100%)	22 (95.7%)
Informal sector	1 (9.1%)	0	1 (4.3%)
Family type			
Nuclear	10 (90.9%)	12 (100%)	22 (95.7%)
Joint	1 (9.1%)	0	1 (4.3%)
Resource person			
Mother	5 (45.4%)	5 (41.6%)	10 (43.5%)
Peer group	1 (9.1%)	3 (25.0%)	4 (17.4%)
Family, friends, teachers	5 (45.4%)	1 (8.3%)	6 (26.1%)
Siblings	0 (0)	1 (8.3%)	1 (4.3%)
Internet	0 (0)	2 (16.6%)	2 (8.7%)
Preferred heath facility			
Government	3 (27.3%)	7 (58.3%)	9 (39.1%)
Private	6 (54.5%)	4 (33.3%)	10 (43.5%)
Local doctors	0	1 (8.3%)	1 (4.3%)
Charitable	1 (9.1%)	0	1 (4.3%)
Both government and private	1 (9.1%)	0	1 (4.3%)
Adolescent clinic			
No knowledge	11 (100%)	12 (100%)	23 (100%)
Visited adolescent clinic			
Never	11 (100%)	12 (100%)	23 (100%)
Substance abuse			
Never	11 (100%)	11 (91.7%)	22 (95.7%)
Chewing tobacco	0 (0)	1 (8.3%)	1 (4.3%)

Lifestyle and health

Healthy Diet and Exercise

The adolescents perceived several foods to be healthy, especially fruits and green leafy vegetables, while junk food was perceived as unhealthy. Exercise was also considered as a key aspect of a healthy lifestyle by some participants.

*“In order to be healthy, one needs to do Meditation, a healthy diet, and avoid junk food”* (P22, 17/Male). *“I think I am slightly obese, so I go running every morning in the nearby area”* (Participant [P] 6, 15/Female).

Participants relied on online media, including the internet, to learn about food and exercise. For instance, one of our participants mentioned: *“I am using Instagram, and there are different channels on that. There is a health channel on which hair, skin, health, yoga, diet, etc., and keeps telling all this*” (P2, 15/Female).

Substance Abuse and Health

The awareness of the harmful effects of tobacco, alcohol, and drug use amongst the adolescents was observed in all the participants, but the accuracy of their knowledge varied considerably. A participant reported a mixed perception of substance abuse: *“I feel this is unhealthy. People may do it within limits. But keeping it within the limit is difficult. This makes their minds stress-free. Once a month, people can have alcohol, but smoking is harmful. Alcohol along with smoking is very harmful”* (P23, 19/Male). In contrast, family episodes of illness resulting from substance abuse reinforced negative attitudes towards their use: *“Cigarette can cause lung or oral cancer, but with alcohol, I don't know. One of my uncles was involved in these drugs, and he expired due to this, so I am a little scared about this”* (P14, 15/Male).

Perception of What Constitutes Good Health

Adolescent boys typically spoke about health in terms of physical wellbeing. The participants assessed their health primarily on their ability to function. When functionality is impaired, health problem arises. For instance, one said: *“Healthy are those who are not having any health problems and unhealthy are those who are having problems”* (P17, 16/Male); another one commented that *“A person who can do daily chores with (full) potential and activeness is healthy”* (P22, 18/Male). Importantly, all participants defined functioning, including physical, mental, and social dimensions of health. The majority of the participants were concerned with body image and size: *“I am healthy as I am. I would like to have more height and less weight”* (P17, 16/Male). Girls preferred a body weight that was proportional to their height, although they were unaware of concepts such as body mass index: *“I look thin, earlier I was very healthy. I don't want to be too fat or too thin”* (P9, 18/Female). *“I don't feel good about my health. I think according to my age, I should be higher and sometimes I feel obese too. I feel I should be a little thinner”* (P2, 18/Female).

Information on Sexual and Reproductive Health

White discharge and severe blood loss were the most common reproductive health concerns among the girls. Girls were taught about menstruation and how to maintain personal hygiene during this period by their teachers. However, when it came to menstrual health concerns, close friends and peers were their first point of contact. 

In the case of boys, all acknowledged that they did not talk to their fathers about reproductive health issues but instead preferred to search on the internet, social media, or query their friends and acquittances but not school teachers: *“I usually search on the internet if I am doubtful for asking my parents. The information from the internet is mostly authentic, but I refer to different sites to get any information. If I see any similar information on a particular topic on multiple websites, it would be true; if there are discrepancies, it will be doubtful. School teachers do not go into deep while teaching this (reproductive health) topic”* (P22, 17/Male). 

Most female adolescents believed that sexual activity should be avoided by young and unmarried women. Family planning is a "taboo" topic among teens. Girls considered mothers as a reliable source of sexual and reproductive health-related information. However, management of the sequela of unplanned sexual encounters was problematic. An adolescent girl narrated an incident:* “One of my friends asked me about abortion pills and all, I didn't get involved or tell anything though I knew about it. I have scolded her saying that you have made mistakes”* (P1, 15/Female). However, some believed that the accessibility to such knowledge was not a challenge: *“As*we* keep growing up, as we go into the situation, we get to know the things by ourselves”* (P2, 15/Female).

The majority of adolescent boys feel that the family planning methods are mainly for females. Some of them reported that they don't know about contraceptives that are available on the market. 

Health seeking behavior

Minor Ailments

The main minor health issues encountered by the participants were skin problems like skin rashes, acne, seasonal flu, fever, etc., for which they preferred either home-based remedies or medicines from local practitioners or pharmacists. Health information was also obtained from internet sources: *"I don’t visit a doctor for common cold or fever. It gets cured on its own, I drink Kadha at home”* (P16, 17/Male). Another participant mentioned: *“When I was suffering from cold, I went to chemist shop and asked for medicine, and I was fine after that”* (P22, 17/Male).

Knowledge About Available Services

All the participants were unaware of the Adolescent Health Services, Adolescent Friendly Health Services, and Adolescent Reproductive and Sexual Health (ARSH) clinics. The girls were aware of a few government programs which they could avail of, including the Weekly Iron/Folic Acid Scheme (WIFS) and the free menstrual pad distribution scheme in government schools. “*I have never heard about adolescence clinic. I have heard it for the first time. But we got iron tablets in the school. Also, we got anti worming tablets from the school once a year”* (P8, 18/Female).

Determinants of Service Utilization

The majority of the participants agreed that treatment in private health facilities was quicker, had minimal waiting time, and the health care providers were available all the time. In contrast, though the government facilities provide free treatment, the waiting time was very long. The fear of COVID-19 infection was also one of the reasons for avoiding government health centers during the lockdown: *“Nowadays, due to coronavirus spread, we are rarely going to the dispensary. There is so much crowd in the dispensary due to covid testing”* (P7, 19/Female).

COVID-19 and adolescents

Awareness About COVID-19

The majority of the participants valued getting information and developing a better grasp of the current situation, and they believed it was critical to keep people informed. All of them reported that they were aware of the recent protocols and the preventive measures: *“We must wear a mask, sanitize our hands, and maintain a distance of two feet in public. We must avoid touching mask repeatedly”* (P13, 18/Male).

Psychosocial Impact

All the individuals found social isolation to be unpleasant and distressing and yearned for the opportunity to maintain contact with family and friends as in pre-pandemic times. Home confinement caused feelings of loneliness, boredom, and depression. Fear of severe COVID-19 disease affecting the parents and elderly was a predominant concern amongst the adolescents: *“We were scared for our parents because they both have some health issues. So, we were worried for them during COVID-19” *(P11, 18/Female).

Educational Impact

In general, participants indicated dissatisfaction with homeschooling at all grade levels and perceived a failure to attain their desired academic goals compared to traditional classroom learning settings. Adolescent students felt that online classes hampered their learning as they were frequently unable to communicate with their teachers and were easily distracted at home: *“COVID-19 affected my education very badly. I am being passed and promoted to the upper class, and I feel I do not know anything; because of this, I will only face the problem. I have forgotten everything I had studied in eighth and ninth classes”* (P19, 15/Male).

Economic Impact

The influence of the pandemic on the economy was examined from the perspective of adolescents. Overall, participants were aware that COVID-19 restrictions caused some family members to lose their jobs or caused a reduction in their wages, which had a negative economic impact on their families.

*“Earlier also there were problems, but now it is worse. My mother is a housewife. My father's factory also got shut down during COVID-19 for a very long time. My maternal uncle is a rickshaw driver; even he was not able to get work during that time”* (P13, 18/Male).

Coping with COVID-19

Participants of all groups reported finding support from their families had reduced their stress. Communicating through internet-based apps also helped. However, there was a substantial increase in adolescent screen time: *“I can use WhatsApp, it has friends and different groups also. I can use Instagram and Facebook, there are some videos on it which I can watch as time pass” *(P2, 15/Female).

## Discussion

This qualitative study explored adolescents' perceptions of health and their health-seeking behavior during the COVID-19 pandemic. Adolescents viewed healthy behaviors such as healthy eating and exercise as important to avoid disease, but it was often a secondary consideration when compared with physical attractiveness, an aesthetic concept. Previous studies have also observed concerns over height and weight, which were more common in adolescents, especially in those reporting psychological problems [12,13]. High-risk behaviors like smoking, drinking, or using soft drugs were frequently identified as expressions of unhealthy behavior and causative agents of various chronic health disorders, although vulnerability to experimentation could not be ruled out due to ambivalent attitudes towards ever use. Previous research has suggested that adolescents are at risk of addiction due to peer pressure, curiosity, and parental addiction [14,15].

Our study findings indicate that adolescents, even in low-income neighborhoods, are becoming increasingly reliant on the internet as a source of health information, especially for sexual and reproductive health problems. Adolescent exposure to television and digital media has been identified as a major factor that influences behavior, including sedentarism [16]. Menstruation was not associated with stigma per se in either gender, but peers rather than teachers or parents were likely to be their sources of information for menstrual health management, a finding in contradiction to some other studies [17]. 

In the present study, young adults had some knowledge about fertility, but it was insufficient to enable them to make informed decisions under compelling circumstances, a finding consistent with studies across South Asia [18]. Most adolescents firmly believe that marriage and childbearing are inextricably tied to sexuality and are inclined to delay their sexual debut. However, these adolescents were uncomfortable discussing sexual concerns with their parents because of the prevalent social taboos. 

The lack of awareness and negligible utilization of specific adolescent health services is suggestive of the primary healthcare system being negligent towards adolescent health concerns. It is important for government health facilities to focus on the branding of AFHSs and conduct routine audits on their service availability and utilization. The government of India's involvement of peer group educators in addressing reproductive and sexual health is a positive step in the right direction, but it requires regular follow-up and monitoring [19].

There is increasing recognition of the detrimental impact of COVID-19, lockdown, and prolonged school closure on adolescent health and development. Adolescents in this study expressed concerns over parental health from potential COVID infections, loss of household income, and lack of academic comprehension through online mode of education. Coping mechanisms of participants to the stress of the ongoing COVID-19 pandemic included greater social media engagement and screentime, a finding similar to that observed in a study amongst Chinese adolescents [20]. 

The strengths of the study include the study setting, which selected a socioeconomically vulnerable population, especially during the period of the COVID-19 pandemic. Face-to-face in-depth interviews were found to be more effective as they helped to understand some knowledge elements that were frequently unexplored while using other traditional methods like focus group discussions. Furthermore, a one-to-one interview represents an unstructured environment enabling exploration and exchange of ideas without having to feel obligated to provide some "correct" response. 

Study limitations

Although this study was conducted in a low-income neighborhood, all adolescents were school-going, and our sample did not include any school drop-outs or delinquent children. Information on questions related to knowledge of HIV and condoms was not collected in this study. Due to the social desirability bias, it is possible for perceived negative behaviors to be underreported. Since the study was conducted after the second wave of the COVID-19 pandemic had subsided in Delhi, recall bias may influence responses related to the pandemic. 

## Conclusions

Adolescents' perceived body image and size considerations apart from functioning at an optimum physical capacity as the principal attributes of good health, which was possible through the intake of a healthy diet and exercise. Awareness of the harmful effects of substance abuse was present, but attitudes were ambivalent, which predisposed them to future risk and experimentation. Most adolescents perceived themselves as lacking access to reliable, adequate, and validated sources of information on sexual and reproductive health. The knowledge and utilization of adolescent health services in their area were negligible, suggesting the need to strengthen those services and improve the program outreach. Future studies should assess adolescents for COVID-19-related depression, anxiety, and stress and assess interventions for promoting healthy coping mechanisms.
